# Supplementary material for: Whole brain delivery of an instability-prone Mecp2 transgene improves behavioral and molecular pathological defects in mouse models of Rett syndrome
Source: eLife. 2020 Mar 24;9:e52629. doi: 10.7554/eLife.52629 (PMC7117907; doi:10.7554/eLife.52629)
Supplement: Supplementary file 1. — Quantitative assessment of transduced (V5+) astrocytes (Sox9+) and neurons (NeuN+) in the cerebral cortical tissue at each different iMecp2 viral dose. [file elife-52629-supp1.docx]

| **Groups (n=3)** | **V5^+^ NeuN^+^/ NeuN^+^** | **V5^+^ NeuN^+^/ V5^+^** | **V5^+^ NeuN^+^/ DAPI^+^** | **V5^+^ Sox9^+^/ Sox9^+^** | **V5^+^ Sox9^+^/ V5^+^** | **V5^+^ Sox9^+^/ DAPI^+^** |
| --- | --- | --- | --- | --- | --- | --- |
| **KO-iMecp2 (10^9^ vg)** | 5,4% ± 6,8% | 26,2% ± 12,7% | 3,4% ± 1,9% | 38,6% ± 12,0% | 36,6% ± 9,3% | 6,2% ± 2,0% |
| **KO-iMecp2 (10^10^ vg)** | 12,1% ± 8,3% | 28,7% ± 13,3% | 7,5% ± 3,5% | 59,5% ± 10,3% | 29,6% ± 8,7% | 9,5% ± 3,5% |
| **KO-iMecp2 (10^11^ vg)** | 50,2% ± 9,4% | 47,6% ± 14,5% | 31,1% ± 2,8% | 55,7% ± 7,5% | 15,4% ± 3,5% | 8,9% ± 1,8% |
| **KO-iMecp2 (10^12^ vg)** | 71,0% ± 12,2% | 64,5% ± 13,1% | 44,6% ± 9,8% | 63,4% ± 9,5% | 14,2% ± 7,3% | 10,1% ± 2,5% |
|  |  |  |  |  |  |  |
| **HET-iMecp2 (10^11^ vg)** | 48,1% ± 16,4% | 42,6% ± 19,1% | 26,4% ± 7,6% | 46,4% ± 7,6% | 7,4% ± 2,9% | 7,4% ± 1,6% |

**Supplementary Table 1.**

Quantitative assessment of transduced (V5^+^) astrocytes (Sox9+) and neurons (NeuN+) in the cerebral cortical tissue at each different iMecp2 viral dose.
