# Supplementary material for: Whole brain delivery of an instability-prone Mecp2 transgene improves behavioral and molecular pathological defects in mouse models of Rett syndrome
Source: eLife. 2020 Mar 24;9:e52629. doi: 10.7554/eLife.52629 (PMC7117907; doi:10.7554/eLife.52629)
Supplement: Supplementary file 2. — Blood serum levels of liver enzyme and liver histochemical analysis (representative images) were used as indicators of liver health. # Reference values for C57BL/6J male mice were taken from the mouse phenome database https://phenome.jax.org/. Abbreviations, Treat: treatment, ALB: albumin, ALP: Alkaline phosphatase, ALT: Alanine aminotransferase, HE: hematoxylin/eosin staining. All values are indicated as mean ± SD, n = 3, SD. Scale bar: 200 µm. [file elife-52629-supp2.docx]

| **Mice** | **Wild-Type** | | **Mecp2^-/y^** | | | **Reference value #** | **Normal Range** |
| --- | --- | --- | --- | --- | --- | --- | --- |
| **AAV**  **(dose)** | None | iMecp2 (10^12^) | None | GFP (10^12^) | iMecp2 (10^12^) | None |  |
| **ALB**  **(g/dL)** | 3,23 ± 0,39 | 3,50 ± 0,69 | 3,17 ± 0,75 | 3,43 ± 0,64 | 3,85 ± 0,35 | 3,87 ± 0,20 | 2,7 / 3,6 |
| **ALP**  **(U/L)** | 109 ± 54 | 116 ± 18 | 131 ± 14 | 114 ± 28 | 140 ± 22 | 140 ± 16 | 100 / 140 |
| **ALT**  **(U/L)** | 43 ± 11 | 52 ± 27 | 71 ± 2 | 60 ± 6 | 68 ± 13 | 79 ± 19 | 0 / 70 |
| **Liver HE** | 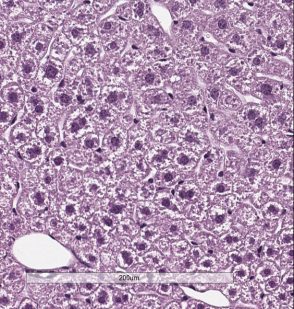 | 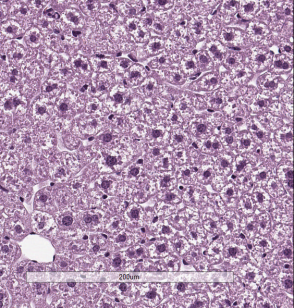 | 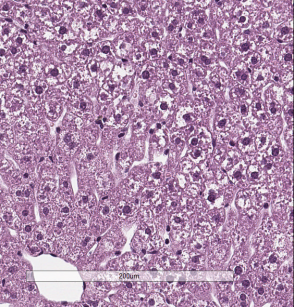 | 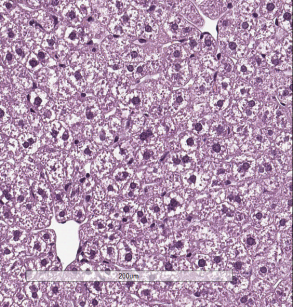 | 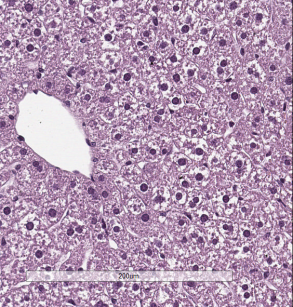 |  |  |

**Supplementary File 1b**

No evidence of liver toxicity in mice administered with high dose of AAV treatments. Blood serum levels of liver enzyme and liver histochemical analysis (representative images) were used as indicators of liver health. # Reference values for C57BL/6J male mice were taken from the mouse phenome database https://phenome.jax.org/. Abbreviations, Treat: treatment, ALB: albumin, ALP: Alkaline phosphatase, ALT: Alanine aminotransferase, HE: hematoxylin/eosin staining. All values are indicated as mean ± SD, n = 3, SD. Scale bar: 200 µm
