# Supplementary material for: Whole brain delivery of an instability-prone Mecp2 transgene improves behavioral and molecular pathological defects in mouse models of Rett syndrome
Source: eLife. 2020 Mar 24;9:e52629. doi: 10.7554/eLife.52629 (PMC7117907; doi:10.7554/eLife.52629)
Supplement: Supplementary file 3. [file elife-52629-supp3.docx]

**Supplementary Table 3**

Primers employed for qRT-PCRs.

| **Name** | **Sequence** |
| --- | --- |
| *Mecp2*-cDNA-endo-F | 5′- ggaggtgtgatgcaggagaa-3′ |
| *Mecp2*-cDNA-endo-R | 5′- tttggattgggttgatgtgg-3′ |
| *Mecp2*-cDNA-ecto-F | 5′-gggagtagttggagcattgg-3′ |
| *Mecp2*-cDNA-ecto-R | 5′-agggatgccacccgtagat-3′ |
| *Mecp2*-cDNA-tot-F | 5′-ccggggacctatgtatgatg-3′ |
| *Mecp2*-cDNA-tot-R | 5′-aagcttttccctggggatt-3′ |
| *β-Actin*-F | 5′-tggcaccacaccttctacaat-3′ |
| *β-Actin*-R | 5′-aggcatacagggacagcaca-3′ |
| 18S-F | 5′- gtaacccgttgaaccccatt-3′ |
| 18S-R | 5′-ccatccaatcggtagtagcg-3′ |
| *Sqle*-F | 5′-aaccaaccaagtgcagagtg-3′ |
| *Sqle*-R | 5′-ccgattacagcatcatcttcc-3′ |
| *Nsdhl*-F | 5′-acatggtggagcagttgctg-3′ |
| *Nsdhl*-R | 5′-cctttgagagctgggtacaggt-3′ |
| *MsmoI*-F | 5′-tgggctgtgcagtcattgagg-3′ |
| *MsmoI*-R | 5′-atggagcctgaaactcgtgat-3′ |
| *Kcnj10-*F | 5′-cgtcggtcgctaaggtctat-3′ |
| *Kcnj10-*R | 5′-gcaatgtgctccattctcac-3′ |
| *Kcnc3-*F | 5′-ggaccgagcttgcttccttg-3′ |
| *Kcnc3-*R | 5′-cgttggcgttgaggtcgg-3′ |
| *Angpl4-*F | 5′-caacgccacccacttaca-3′ |
| *Angpl4-*R | 5′-aatcactgtccagcctccat-3′ |
| *Mecp2-*gDNA-F | 5′-agaaagcctgggagtgtggt -3′ |
| *Mecp2-*gDNA-R | 5′-cttccttgacctcgatgctg -3′ |
| *Lmnb2*-gDNA-F | 5′-gttaacactcaggcgcatgggcc-3′ |
| *Lmnb2*-gDNA-R | 5′-ccatcagggtcacctctggttcc-3′ |
