# Supplementary material for: Whole brain delivery of an instability-prone Mecp2 transgene improves behavioral and molecular pathological defects in mouse models of Rett syndrome
Source: eLife. 2020 Mar 24;9:e52629. doi: 10.7554/eLife.52629 (PMC7117907; doi:10.7554/eLife.52629)
Supplement: Supplementary file 4. [file elife-52629-supp4.docx]

**Supplementary Table 4**

**List of antibodies for flow cytometry.**

| **Reactivity** | **Fluorophore** | **Clone** | **Company** |
| --- | --- | --- | --- |
| Mouse | PE-CD3 | 17A2 | BD Pharmingen |
| Mouse | PE-Cy7-CD4 | GK1.5 | E-Bioscence |
| Mouse | FITC-CD8a | 53-6.7 | BD Pharmingen |
| Mouse | APC-Cy7-CD44 | IM7 | BD Pharmingen |
| Mouse | BV786-CD62l | Mel-14 | BD Pharmingen |
