# Supplementary material for: Whole brain delivery of an instability-prone Mecp2 transgene improves behavioral and molecular pathological defects in mouse models of Rett syndrome
Source: eLife. 2020 Mar 24;9:e52629. doi: 10.7554/eLife.52629 (PMC7117907; doi:10.7554/eLife.52629)
Supplement: Transparent reporting form [file elife-52629-transrepform.pdf]

eLIFE
1st Floor
24 Hills Road
Cambridge CB2 1JP, UK
P 01223 855340
W elifesciences.org
T @elife

eLife's transparent reporting form

We encourage authors to provide detailed information *within their submission* to facilitate the interpretation and replication of experiments. Authors can upload supporting documentation to indicate the use of appropriate reporting guidelines for health-related research (see EQUATOR Network), life science research (see the BioSharing Information Resource), or the ARRIVE guidelines for reporting work involving animal research. Where applicable, authors should refer to any relevant reporting standards documents in this form.

If you have any questions, please consult our Journal Policies and/or contact us: editorial@elifesciences.org.

Sample-size estimation

- You should state whether an appropriate sample size was computed when the study was being designed
- You should state the statistical method of sample size computation and any required assumptions
- If no explicit power analysis was used, you should describe how you decided what sample (replicate) size (number) to use

Please outline where this information can be found within the submission (e.g., sections or figure legends), or explain why this information doesn't apply to your submission:

At the planning stage, we based our expectations on similar experiments previously performed in our laboratory, as well as in the literature. For *in vitro* analysis 3 or more biological replicates were included. The number of biological replicates used for each experiment is specified in the figure legends. For the experimental cohorts, we used 5 to 14 mice per genotype per dose. The number of animals used for each experiment is specified in the figure legends. For histological, biochemical and transcriptomic studies, 3 or more samples were used for analysis, as indicated in the figure legends.
Biological replicates of the experiments are a necessity of this kind of study, as typically only a small number of mice with the appropriate genotype (Mecp2$^{-/y}$ male or Mecp2$^{+/-}$ female) are born in each litter. Control littermates animals were included in each of the biological replicates.
Each experiment typically included multiple analysis time points, and a similar number of experimental and control animals were included in each of those.

Replicates

- You should report how often each experiment was performed
- You should include a definition of biological versus technical replication
- The data obtained should be provided and sufficient information should be provided to indicate the number of independent biological and/or technical replicates
- If you encountered any outliers, you should describe how these were handled
- Criteria for exclusion/inclusion of data should be clearly stated
- High-throughput sequence data should be uploaded before submission, with a private link for reviewers provided (these are available from both GEO and ArrayExpress)

Please outline where this information can be found within the submission (e.g., sections or figure legends), or explain why this information doesn't apply to your submission:

In vivo experiments were performed including at least 3 mice for groups, considering treated and control. These experiments were repeated at least two independent times to reach the minimal amount of 5 animals for represented cohorts. In rare cases we excluded mice from the cohorts because of defects independent from the phenotype (such as malformations of the maxillary incisors).
In vitro experiment and other analysis (histological, biochemical, transcriptomical) were performed once time including at least 3 biological replicates or 2-3 times including more than 3 biological replicates. Dots plot and figure legends are comprehensive of the biological replicated used.
Technical replicates are not presented as single values in figures, only biological replicates were graphed. Each dot in the graphs represents a biological replicate and the analysis was performed at least with two technical replicates.
We considered random noise outliers below 5th or above 95th percentile.

Statistical reporting

- Statistical analysis methods should be described and justified
- Raw data should be presented in figures whenever informative to do so (typically when N per group is less than 10)
- For each experiment, you should identify the statistical tests used, exact values of N, definitions of center, methods of multiple test correction, and dispersion and precision measures (e.g., mean, median, SD, SEM, confidence intervals; and, for the major substantive results, a measure of effect size (e.g., Pearson's r, Cohen's d)
- Report exact p-values wherever possible alongside the summary statistics and 95% confidence intervals. These should be reported for all key questions and not only when the p-value is less than 0.05.

Please outline where this information can be found within the submission (e.g., sections or figure legends), or explain why this information doesn't apply to your submission:

Specific statistical methods used in each experiment are detailed in the figure legends. Raw data are presented in most figures with each sample represented by an individual dot, instead in time-course analysis only the means and the SD of the measurement is presented. Specific p-values for the graphs are indicated with symbols explained in the figure legends as well as the statistical test used. Moreover statics are detailed in materials and methods section.

(For large datasets, or papers with a very large number of statistical tests, you may upload a single table file with tests, Ns, etc., with reference to sections in the manuscript.)

Group allocation

- Indicate how samples were allocated into experimental groups (in the case of clinical studies, please specify allocation to treatment method); if randomization was used, please also state if restricted randomization was applied
- Indicate if masking was used during group allocation, data collection and/or data analysis

Please outline where this information can be found within the submission (e.g., sections or figure legends), or explain why this information doesn't apply to your submission:

Groups were allocated according to genotype (*Mecp2*$^{-/y}$ and *Mecp2*$^{+/y}$ male, *Mecp2*$^{+/-}$ and *Mecp2*$^{+/+}$ female), viral dose (10^{9-12} vg/mouse), transgene (*MECP2*, *Mecp2* or GFP) and immunosuppressive treatment (± Ciclosporine, Csa). The specific grouping for each figure is indicated in the corresponding test, legend and/or methods section.

Additional data files ("source data")

- We encourage you to upload relevant additional data files, such as numerical data that are represented as a graph in a figure, or as a summary table
- Where provided, these should be in the most useful format, and they can be uploaded as "Source data" files linked to a main figure or table
- Include model definition files including the full list of parameters used
- Include code used for data analysis (e.g., R, MatLab)
- Avoid stating that data files are "available upon request"

Please indicate the figures or tables for which source data files have been provided:

Raw data relative to transcriptomic analysis presented in figure 6 have been deposited in the NCBI GEO repository with the accession number GSE125155, accessible with the reviewer token yfcpaescpbwpdgj.
